# Implementation strategies in the Exploration and Preparation phases of a colorectal cancer screening intervention in community health centers

**DOI:** 10.1186/s43058-023-00485-5

**Published:** 2023-09-20

**Authors:** Renée M. Ferrari, Jennifer Leeman, Alison T. Brenner, Sara Y. Correa, Teri L. Malo, Alexis A. Moore, Meghan C. O’Leary, Connor M. Randolph, Shana Ratner, Leah Frerichs, Deeonna Farr, Seth D. Crockett, Stephanie B. Wheeler, Kristen Hassmiller Lich, Evan Beasley, Michelle Hogsed, Ashley Bland, Claudia Richardson, Mike Newcomer, Daniel S. Reuland

**Affiliations:** 1 Lineberger Comprehensive Cancer Center, University of North Carolina at Chapel Hill, 450 West Drive, Chapel Hill, NC 27599 USA; 2 Gillings School of Global Public Health, University of North Carolina at Chapel Hill, 135 Dauer Drive, Chapel Hill, NC 27599 USA; 3 School of Nursing, University of North Carolina at Chapel Hill, 120 North Medical Drive, Chapel Hill, NC 27599 USA; 4 School of Medicine, University of North Carolina at Chapel Hill, 5034 Old Clinic Building, Chapel Hill, NC 27599 USA; 5 UNC Institute for Healthcare Quality Improvement, CB #8005, Chapel Hill, NC 27599 USA; 6 College of Health and Human Performance, East Carolina University, 2307 Carol G. Belk Building, Greenville, NC 27858 USA; 7 Division of Gastroenterology & Hepatology, Oregon Health & Science University, 3161 SW Pavilion Loop, Portland, OR 97239 USA; 8 Blue Ridge Health, UNC Health, 2579 Chimney Rock Road, Hendersonville, NC 28792 USA; 9 Blue Ridge Health, 2759 Chimney Rock Road, Hendersonville, NC 28792 USA; 10 Ahoskie Comprehensive Care, Roanoke Chowan Community Health Center, 120 Health Center Drive, Ahoskie, NC 27910 USA; 11 Digestive Health Partners, 191 Biltmore Avenue, Asheville, NC 28801 USA; 12 Western Carolina Medical Society, 304 Summit Street, Asheville, NC 28803 USA

**Keywords:** Cancer screening, Colorectal cancer, Community health centers, Fecal immunochemical test, Patient navigation, Vulnerable populations, Implementation, Exploration, Preparation, Framework

## Abstract

**Background:**

Adoption of colorectal cancer (CRC) screening has lagged in community health center (CHC) populations in the USA. To address this implementation gap, we developed a multilevel intervention to improve screening in CHCs in our region. We used the Exploration, Preparation, Implementation, Sustainment (EPIS) framework to guide this effort. Here, we describe the use of implementation strategies outlined in the Expert Recommendations for Implementing Change (ERIC) compilation in both the Exploration and Preparation phases of this project. During these two EPIS phases, we aimed to answer three primary questions: (1) What factors in the inner and outer contexts may support or hinder colorectal cancer screening in North Carolina CHCs?; (2) What evidence-based practices (EBPs) best fit the needs of North Carolina CHCs?; and (3) How can we best integrate the selected EBPs into North Carolina CHC systems?

**Methods:**

During the Exploration phase, we conducted local needs assessments, built a coalition, and conducted local consensus discussions. In the Preparation phase, we formed workgroups corresponding to the intervention’s core functional components. Workgroups used cyclical small tests of change and process mapping to identify implementation barriers and facilitators and to adapt intervention components to fit inner and outer contexts.

**Results:**

Exploration activities yielded a coalition of stakeholders, including two rural CHCs, who identified barriers and facilitators and reached consensus on two EBPs: mailed FIT and navigation to colonoscopy. Stakeholders further agreed that the delivery of those two EBPs should be centralized to an outreach center. During Preparation, workgroups developed and refined protocols for the following centrally-delivered intervention components: a registry to identify and track eligible patients, a centralized system for mailing at-home stool tests, and a process to navigate patients to colonoscopy after an abnormal stool test.

**Conclusions:**

This description may be useful both to implementation scientists, who can draw lessons from applied implementation studies such as this to refine their implementation strategy typologies and frameworks, as well as to implementation practitioners seeking exemplars for operationalizing strategies in early phases of implementation in healthcare.

**Supplementary Information:**

The online version contains supplementary material available at 10.1186/s43058-023-00485-5.

Contributions to the literature

- Adds to limited empiric research focused on the Exploration and Preparation phases of the Exploration, Preparation, Implementation, Sustainment (EPIS) framework in developing a multilevel intervention.
- Demonstrates how implementation strategies can be operationalized during the Exploration and Preparation phases, drawing upon methods from both improvement and systems science.
- Details how implementation strategies used during the Exploration phase led to a key decision to centralize the delivery of intervention components within a large intermediary organization rather than solely within practice settings.
- Illustrates how implementation strategies were used to develop bridging factors (factors that link inner and outer contexts, e.g., community-academic partnerships) to coordinate and support interactions across the multiple organizations involved in implementation.
- Identifies two strategies, not currently listed in the Expert Recommendations for Implementing Change (ERIC) compilation, that could be considered discrete implementation strategies: review and appraise current evidence regarding effective interventions; and use systems science tools to map and model complex processes.

## Background

Despite strong evidence that colorectal cancer (CRC) screening reduces mortality, screening remains underused in the USA [1, 2]. Screening rates are especially low in people who are medically underserved and those without health insurance [2–4]. As in many US regions, community health centers (CHCs) in North Carolina serve diverse populations, including many with lower incomes and/or who lack health insurance. Although screening rates in populations served by North Carolina (NC) CHCs have increased in recent years, they remain below state and national averages [5].

To address this CRC screening implementation gap, we initiated the Scaling Colorectal Cancer Screening through Outreach, Referral, and Engagement (SCORE) project [6]. Our overall approach for developing the project was grounded in the Institute for Healthcare Improvement’s (IHI) Improvement Model [7], a widely-used model familiar to our CHC partners. The Improvement Model provides guidance and tools (e.g., process maps, Plan-Do-Study-Act cycles) to iteratively plan and test improvements in care delivery. We also applied the Exploration, Preparation, Implementation, Sustainment (EPIS) framework [8] to guide planning and implementation of a complex, multilevel CRC screening intervention.

In this paper, we describe the implementation strategies used during the Exploration and Preparation phases of EPIS and the multilevel determinants that guided design of those strategies. The Exploration phase of EPIS involves engaging stakeholders, selecting evidence-based practices (EBPs), and identifying needs, opportunities, and challenges at the levels of the practice setting and wider context. The Preparation phase entails identifying and planning for barriers and facilitators related to the selected EBP(s). The EPIS framework further identifies factors that may impede or facilitate implementation at the level of the outer context factors (e.g., inter-organizational networks), inner context (e.g., organizational characteristics), bridging factors, and the innovation being implemented [8]. Bridging factors are a relatively new addition to the EPIS framework and include relational ties, formal arrangements, and other factors that connect the outer and inner contexts [9]. To the extent possible, strategies were aligned with terminology from the Expert Recommendations for Implementing Change (ERIC) compilation of implementation strategies [10]. To our knowledge, few studies have provided a detailed focus on the application of ERIC strategies to illustrate empirically how implementation strategies may be operationalized and adapted during the Exploration and Preparation phases of a complex implementation study [11].

During the Exploration and Preparation phases of the SCORE project, we aimed to answer three primary questions: (1) What factors in the inner and outer contexts may support or hinder colorectal cancer screening in North Carolina CHCs?; (2)What EBPs best fit the needs of North Carolina CHCs?; and (3) How can we best integrate the selected EBPs into North Carolina CHC systems?

## Methods

This study was conducted as part of the NCI-funded consortium The Accelerating Colorectal Cancer Screening and Follow-up through Implementation Science (ACCSIS) Program. The overall aim of ACCSIS is to conduct multi-site, coordinated, transdisciplinary research to evaluate and improve CRC screening processes using implementation science. Activities described here were largely conducted between October 2016 and March 2020. Of note, much of the Exploration phase was conducted prior to study funding in 2018, as part of our university cancer center’s commitment to reducing cancer burden in North Carolina. ACCSIS funding provided the means to complete the Exploration phase and progress through the other EPIS phases.

We present methods and results by EPIS phase — first describing methods and results for the Exploration phase and then for the Preparation phase of the project. We began using the EPIS framework early in the study when we realized how well it aligned with the work we were doing. At that point, we categorized identified barriers and facilitators using EPIS constructs and then applied EPIS moving forward. We used the implementation strategy reporting guidelines recommended by Proctor and colleagues to guide the reporting of our work [12]; details are provided as an Additional file.

## Exploration phase methods

The Exploration phase involved three key implementation strategies, summarized in Table 1 and described below.

**Table 1:** Key SCORE implementation strategies, definitions, and outcomes used during the Exploration phase

**Exploration phase aim:** *evaluate community needs and intervention fit*						
**Strategies**	**ERIC definition**	**Actor**	**Action** **SCORE operationalization**	**Action target**	**Temporality**	**Outcomes**
1. Conduct local needs assessment	Collect and analyze data related to the need for the innovation	Research team	Conduct a literature review and a secondary data analysis of epidemiological data specific to NC, and a systematic review and meta-analysis of CRC screening interventions	State of NC Partner CHC regions	Prior to funding and during the early stages of the Exploration phase	• Identified colorectal cancer hotspot • Identified inequities (e.g., insurance status, rurality, proximity to colonscopy services) • Identified strong evidence supporting mailed FIT outreach • Identified evidence supporting navigation in promoting colonoscopy completion
2. Build a coalition	Recruit and cultivate relationships with partners in the implementation effort	Research team	Engage cancer center leadership, the NC CRC Roundtable, others across state working to increase CRC screening, including endoscopy centers, and CHC stakeholders	Cancer center leadership; NC CRC Roundtable; NC Society for Gastroenterology; gastroenterologists across the state; CHCs ready and willing to partner around a CRC screening intervention	During late stages of Exploration, after ACCSIS funding received	• Cancer center leadership committed resources • Established partnerships with two CHCs with diverse populations • Established partnerships with colonoscopy providers in the western region and relationships with providers in the eastern region • Established stakeholder relationships with NC CRC Roundtable and members of the NC Society for Gastroenterology
3. Conduct local consensus discussions	Include local providers and other stakeholders in discussions that address whether the chosen problem is important and whether the clinical innovation to address it is appropriate	Research team and community health center administrators including executive directors, medical directors, and practice managers	Develop consensus about criteria to guide intervention design and implementation based on needs assessment and stakeholder input	Research team and CHC administrators and leadership	During Exploration, after partner CHCs identified	• Developed guiding criteria for the intervention: (1) act across the screening care continuum and at multiple levels; (2) account for CHC staff time constraints; (3) focus on non-visit-based (outreach) approaches to screening as a complement to visit-based screening; (4) facilitate follow-up colonoscopy for an abnormal FIT; (5) be replicable across multiple CHCs with varying context

### Exploration strategy 1: conduct local needs assessment

As noted previously, the research team has a long-standing interest in CRC screening in NC and began to assess needs prior to initiation of the SCORE project. Early needs assessment included review of existing data and the literature on CRC burden in North Carolina as well as review of the literature on CRC screening interventions. To identify the EBPs that would likely have the most impact in our setting, we also assessed the evidence regarding interventions for CRC screening effectiveness. This included conducting a systematic review and meta-analysis of randomized trials assessing evidence-based interventions to increase CRC screening [13].

To assess the burden of CRC in North Carolina, members of the research team conducted a PubMed search of epidemiologic studies [14] and consulted experts in geospatial methods [15] to assess the distribution of CRC mortality and morbidity in North Carolina. Based on these findings, the research team engaged with a CHC in a northeastern region of the state that was identified as a CRC “hotspot” based on its high level of CRC burden. This CHC served a predominantly Black population. To assess needs across diverse contexts, the team also engaged with a CHC in western NC that served a predominantly White population, a large proportion of whom are Latino. Finally, the team engaged with a third CHC in the southern region of the state that serves a largely Native American patient population.

The team engaged the three CHCs in identifying and prioritizing inner and outer contextual factors salient to CRC screening in NC. In this early phase of the study, engagement involved relationship-building and exploration rather than formal engagement methods. Engagement occurred through site visits, attendance at standing CHC meetings (sometimes presenting information and seeking input), and through ongoing discussions.

The research team approached CHCs with humility and the understanding that CHCs are experts in their own context, participating in open, honest, transparent conversations in which each entity was valued as equal members. Over multiple discussions, the research team shared data from the needs assessment and the CHCs shared successes and challenges with CRC screening specific to their context. Prior to each encounter, the research team and CHC leaders would prioritize discussion topics. For site visits, formal agendas were created. Discussions were led by the principal investigator or project director, depending on the context. In all engagements with stakeholders, we aimed for open discussions, welcoming and carefully considering all opinions and thoughts.

### Exploration strategy 2: build a coalition

The research team built a coalition of stakeholders that would serve as a primary bridging factor linking outer and inner contexts. In keeping with ERIC terminology, we defined “coalition” as “relationships with partners in our implementation efforts.” Early in the Exploration phase and prior to study funding, we developed relationships with key stakeholders working to improve CRC screening, which we then expanded. During semi-annual meetings of the North Carolina Colorectal Cancer Roundtable, we invited persons to engage in discussions about CRC screening. The coalition included the North Carolina Society for Gastroenterology, state Division of Public Health’s Cancer Prevention and Control Branch, North Carolina Colorectal Cancer Roundtable [16], North Carolina Community Health Center Association [17], and regional programs and health care providers who had worked to improve colonoscopy access for the uninsured. Because colonoscopy is required for follow-up after an abnormal FIT-based screening test, and CHCs do not provide colonoscopy services, we also identified and engaged endoscopy providers. In conversations with stakeholders, we identified existing initiatives and resources and began discussing ideas for building on existing efforts across the state and within community health centers.

### Exploration strategy 3: conduct local consensus discussions

**Fig. 1:**
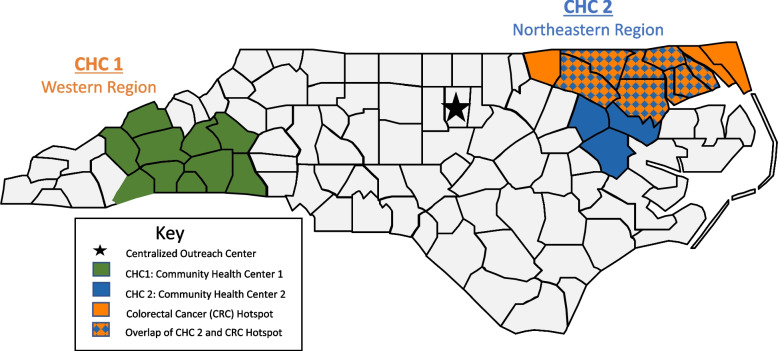
SCORE study sites

Two of the three CHCs were willing and ready to partner with us on all four EPIS phases (Fig. 1). Collectively, these two CHCs operate 17 total clinic sites. The population served by CHC1 is largely rural, White (63%), and Hispanic (29%); about half are uninsured (56%) [18]. CHC2 serves a rural, largely African American (59%) or White (39%) population, 86% of whom are insured through Medicare, Medicaid, or private insurers [18].

The research team made additional site visits and scheduled teleconferences with administrators and practice managers at each of these CHCs to map current CRC screening practices and workflows within their systems. Together, we developed a shared understanding of current practices and existing barriers to CRC screening. The research team shared the latest scientific evidence about CRC screening and findings from their prior CRC screening intervention research [4, 13, 19, 20]. We collaboratively discussed candidate EBPs with respect to their potential effectiveness, feasibility, and potential to complement current screening efforts, and together developed consensus (verbal agreement of the research team and CHC administrators) on the EBPs that would be most effective in this context.

## Exploration phase results

Exploration phase results include (1) identification of multilevel barriers and facilitators to CRC screening in North Carolina CHCs, (2) consensus on criteria for selecting EBPs, and (3) selection of EBPs that fit criteria.

### Multilevel barriers and facilitators

**Fig. 2:**
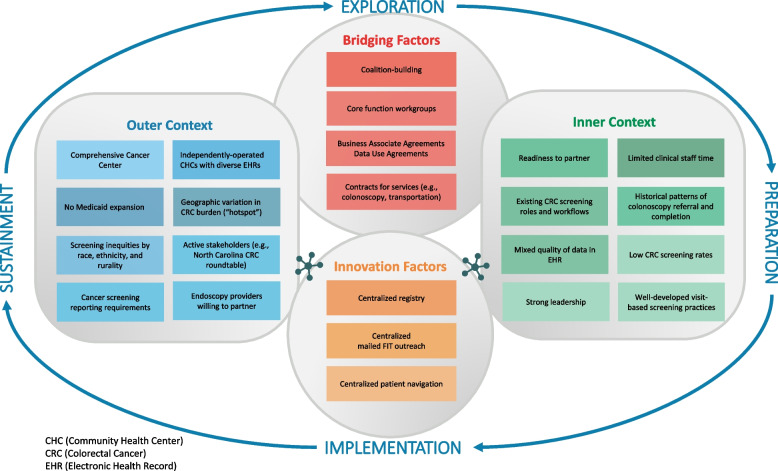
Modified EPIS framework adapted for SCORE project

Review of the literature and ongoing discussions during meetings with stakeholders, including statewide roundtable meetings, helped to identify CRC screening barriers and facilitators (Fig. 2). At the level of the outer context, barriers included the state’s large number of uninsured and lack of Medicaid expansion; racial, income, and rural inequities in screening rates [15]; and 40 CHCs that are independently operated with diverse electronic health records (EHRs). Facilitators included our university’s comprehensive cancer center, which serves the entire state, and active stakeholder partners, including both CHC and endoscopy providers. At the level of the inner context, barriers included mixed quality of patient data in the EHR and constraints on clinic staff time [21–23]. Facilitators included CHCs’ existing CRC screening roles and workflows, strong leadership, and well-developed visit-based screening practices. Barriers and facilitators identified during Exploration were similar across the two CHCs participating in the study, with the exception of access to colonoscopy. In CHC1, a local provider had developed a network of endoscopists willing to provide low-cost colonoscopies to the uninsured; no such network existed for CHC2.

### Criteria for selecting EBPs

The research team met, discussed, and identified a set of criteria for selecting EBPs based on what was learned from the Exploration phase needs assessment, local consensus discussions with stakeholders, and our own work in this field. Table 2 provides an overview of how criteria align with factors in the inner and outer contexts. The criteria stipulated that the intervention should: (1) act across the screening care continuum (e.g., primary care, endoscopy specialty care) and at multiple levels (patient, provider, and system); (2) account for clinical staff time constraints in primary care; (3) focus on non-visit-based (outreach) approaches to screening that would complement existing visit-based screening efforts; (4) facilitate follow-up colonoscopy after an abnormal FIT; and (5) be replicable and adaptable across multiple CHCs with varying contexts.

**Table 2:** Criteria and contextual factors that guided selection of CRC screening EBPs

**Criteria**	**Contextual factors**
1. The intervention should act across the screening care continuum and at multiple levels	• CRC screening is a complex process involving several steps along a continuum of care • Screening includes identifying eligible populations to screen, diagnostic follow-up, and subsequent treatment and surveillance • Multiple patient-, provider-, and system-level factors affect how and if patients move through each step • This complexity suggests that interventions operating only at a single point along the screening continuum or at a single level would have limited impact on screening [24]
2. The intervention should account for clinical staff time constraints in primary care	• The inner context of CHCs is characterized by substantial clinician and staff time constraints • Time constraints represent a major barrier to delivering guideline-recommended clinical services, including CRC screening [21–23]
3. The intervention should focus on non-visit-based (outreach) approaches to screening	• Partner CHCs had made significant progress in improving CRC screening among patients who attended regular clinic visits, maximizing screening rates using visit-based approaches • Visit-based approaches to screening are missing the sizeable number of patients who visited the clinic infrequently or only when acutely ill
4. The intervention should facilitate follow-up colonoscopy after abnormal FIT	• Most CHC providers were using FIT-based screening • Effective FIT-based screening requires that individuals with abnormal stool tests undergo follow-up colonoscopy • Partner CHCs reported financial, transportation, and other barriers to completing these follow-up colonoscopies, consistent with published evidence of low rates of colonoscopy completion after abnormal FIT for socio-economically challenged populations [25] • Endoscopy access is limited and varied across regions • Facilitating colonoscopy completion for the uninsured was seen as especially important as North Carolina is one of 12 states that has not, as of this writing, implemented Medicaid expansion, contributing to high rates of uninsured [26] • One of our partner CHCs serves a large undocumented immigrant population that is ineligible for Medicaid or federal health insurance subsidies • Partner CHCs had limited navigation support for FIT + patients
5. The intervention should be replicable and adaptable across multiple CHCs to address service fragmentation and reduce process variation	• NC CHCs are not integrated, operate independently of each other, and use a variety EHR systems, fecal test kits, clinical laboratories, and independent endoscopy providers • CHCs serve patients with varying insurance types, including government, private, and no insurance

*CHC* Community health center, *CRC* Colorectal cancer, *EHR* Electronic health record, *FIT* Fecal immunochemical test, *NC* North Carolina, *SCORE* Scaling Colorectal Cancer Screening through Outreach, Referral,  and Engagement

### Selection of EBPs

Exploration phase consensus discussions culminated in agreement to move forward with two evidence-based practices: mailed FIT and patient navigation.

Based on prior review of the CRC screening literature [13], the research team identified mailed FIT and patient navigation as CRC EBPs that aligned with the selection criteria. Mailed FIT has potential to reach patients outside the clinic visit; patient navigation addresses barriers to accessing colonoscopies following a positive FIT; and combining the two EBPs reaches patients across the screening care continuum.

To address the two final criteria, staff time constraints and replicability across CHCs, the decision was made to centralize the delivery of mailed FIT and navigation within an intermediary organization (an NCI-designated Comprehensive Cancer Center) rather than within the individual CHCs. This decision enabled us to address constraints on the time staff had available to implement mailed outreach. Centralization also had potential to address the high degree of screening service fragmentation (i.e., poor integration among primary care clinics and endoscopy centers that sometimes led to poor follow-up on endoscopy referrals). Finally, centralization provided opportunities to reduce screening process variation across CHCs with diverse EHRs and barriers to colonoscopy (e.g., access to endoscopists).

We agreed to create a centralized outreach center that would perform the intervention’s core functions (basic purposes of an intervention) [27]. To determine the core functions, we first identified the multiple clinical steps and supports needed along the CRC screening pathway. We then grouped them according to their overall function (purpose), ensured the groupings were mutually exclusive, and settled on three core functions for SCORE: identify and track screening-eligible patients, mail FIT kits, and navigate patients to follow-up colonoscopy. Despite this centralization of infrastructure support to an outreach center, the CHCs remained the patient’s point of care, providing data on eligible patients, reinforcing the importance of CRC screening, assisting with navigation to follow-up colonoscopy, and documenting test results and follow-up in the EHR.

Figure 2 depicts our conceptualization in a modified version of the EPIS framework, showing these centralized functions as innovation factors that interact with outer context, inner context, and bridging factors. The elements of our modified framework emerged over time in response to our project activities and findings. For example, while we identified inner and outer contextual factors early on in Exploration, the concept of centralization arose later following consensus discussions about how to implement the intervention.

## Preparation phase methods

During the Preparation phase, we aimed to understand how best to implement the EBPs collectively identified during Exploration. To do so, the SCORE project used nine key implementation strategies to further develop the intervention and plan for its implementation (Table 3).

**Table 3:** Key SCORE implementation strategies, definitions, and outcomes used during the Preparation phase

**Preparation phase aim:** *plan how to integrate intervention into system*						
**Strategies**	**ERIC definition**	**Actor**	**Action** **SCORE operationalization**	**Action target**	**Temporality**	**Outcomes**
1. Use advisory boards and workgroups	Create and engage a formal group of multiple kinds of stakeholders to provide input and advice on implementation efforts and to elicit recommendations for improvements	Research team and CHC administrators, providers, and staff	Establish workgroups corresponding to CRC screening steps and comprising relevant stakeholders	Workgroups at CHC1 and CHC2	Throughout Preparation	• Established three workgroups to carry out three core functions: (1) Registry (research staff with EHR expertise, CHC data programmers); (2) Mailed FIT (research staff with community-building and implementation expertise, CHC practice managers, medical officers, referral staff); (3) FIT + to Colonoscopy (research staff project manager, patient navigator, internal medicine physician, gastroenterologists, CHC medical director) • Developed workgroup charters to guide workgroup purpose and activities
2. Obtain formal commitments	Obtain written commitments from key partners that state what they will do to implement the innovation	Research team	Obtain (1) commitments from working group members; (2) regulatory and legal agreements to conduct screening outreach and follow-up on behalf of the clinics	Workgroup members of CHC1 and CHC2; CHC1 and CHC2 administration; cancer center leadership; university regulatory, legal, and financial entites; lab results processesing facilities	Workgoup member agreements obtained at the beginning of Preparation. Remaining commitments obtained throughout Preparation, completed before (and required as part of) Implementation	• Workgroup charters detailing workgroup expectations and commitment • Business Associate Agreements to send FITs and conduct follow-up on behalf of the clinics and enable EHR access • DUA entailing data sharing plans for the transfer of data between centralized outreach center and each CHC • EHR access for centralized patient navigator
3. Assess for readiness and identify barriers and facilitators	Assess various aspects of an organization to determine its degree of readiness to implement, barriers that may impede implementation, and strengths that can be used in the implementation effort	Research team and CHC workgroups	Conduct site visits to CHCs and endoscopy centers to understand available resources and capacity; engage CHCs in process mapping and intervention planning discussions; conduct clinic-level chart reviews to inform understanding of referral practices	CHC1 and CHC2	Throughout Preparation	• Multiple visits to CHCs and endoscopy practices, discussing referral protocols and workflows • Process maps of current processes and planned implementation processes •Chart review outcomes • Intervention protocols reflecting involved organizations’ readiness, barriers, and facilitators
4. Conduct cyclical small tests of change	Implement changes in a cyclical fashion using small tests of change before taking changes system-wide. Tests of change benefit from systematic measurement, and results of the tests of change are studied for insights on how to do better. This process continues serially over time, and refinement is added with each cycle	Research team and workgroup members	Within each workgroup, pilot intervention components and test them using PDSA cycles, to iteratively improve SCORE components; incorporate patient feedback	Research team, workgroup members at CHC1 and CHC 2	During Preparation after process mapping	• EHR query validated against manual patient-level chart review • Lab order requisition form tailored to SCORE and each CHC • MailedFIT packaging materials revised following return response • Patient primer letter alerting to expect FIT kit in mail revised following patient input • 4-call navigation protocol adapted from 6-call protocol • Patient-facing navigation materials tailored to patient needs
5. Use data warehousing techniques	Integrate clinical records across facilities and organizations to facilitate implementation across systems	Research team and workgroup members with EHR expertise, including CHC data management staff	Gain CHC EHR access for centralized outreach staff; develop screening registry to accurately and efficiently conduct and track screening activities and support monitoring and reporting, using input from each workgroup’s PDSA cycles	Research team and data management staff at CHC1 and CHC2	Early in Preparation, and before initiating pilot mailedFIT and navigation cycles	• Secure, integrated CRC registry database of patient-level data from different sources (CHCs, endoscopy centers, and navigator calls)
6. Develop educational materials	Develop and format manuals, toolkits, and other supporting materials in ways that make it easier for stakeholders to learn about the innovation and for clinicians to learn how to deliver the clinical innovation	Research team and workgroup members	Develop SOP and supporting materials for implementers and patients within the Registry, MailedFIT, and FIT + to Colonoscopy workgroups to raise awareness, educate, and guide implementation communications (inform using stakeholder feedback and patient interviews); develop process maps to depict intervention workflows	SOP target is research team; Process maps target is research team and workgroup members at CHC1 and CHC2	Throughout Preparation, completed prior to Implementation	• Functional, detailed SOP with supporting materials and process maps for each workgroup/implementer, including site-specific query and intervention tracking protocols (Registry workgroup); protocols for assembling FIT kits; patient-facing materials including primer letter, FIT instructions, and results notification (MailedFIT Workgroup); navigator protocols for patient navigation and questions, and patient-facing materials including welcome to navigation letter, bowel prep instructions, unable to reach and declined navigation letters (FIT + to Colonoscopy Workgroup)
7. Fund and contract for the clinical innovation	Governments and other payers of services issue requests for proposals to deliver the innovation, use contracting processes to motivate providers to deliver the clinical innovation, and develop new funding formulas that make it more likely that providers will deliver the innovation	Research team	Subcontract for specific services to fund laboratory testing of FIT samples and FIT + to colonoscopy activities (transportation, bowel prep, colonoscopy, interpreter services)	Transportation programs and pharmacy services at CHC1 and CHC2; gastroenterology providers at referral clinics; interpreter service providers serving each CHC	Early in Preparation, for completion prior to Implementation	• Contract for flat, reduced-fee colonoscopies for uninsured patients at CHC1, as an extension of an existing program • Contract with CHC2 to fund transportation services as part of an existing CHC transportation program • Payment systems to fund colonoscopies and related services
8. Develop a formal implementation blueprint	Develop a formal implementation blueprint that includes all goals and strategies. The blueprint should include the following: (1) aim/purpose of the implementation; (2) scope of the change (*e.g*., what organizational units are affected); (3) timeframe and milestones; and (4) appropriate performance/progress measures. Use and update this plan to guide the implementation effort over time	Research team and workgroup members	Develop SOPs and process maps corresponding to each CRC screening step and update as needed	Research team and CHC1 and CHC2 leadership and workgroup members	Throughout Preparation, for completion prior to Implementation	• Written SOP outlining processes to guide implementation • Process maps to formalize blueprint for who would do what and when during the Implementation phase

*CHC* Community health center, *CHC1* Community health center 1, *CHC2* Community health center 2, *CRC* Colorectal cancer, *DUA* Data Use Agreement, *EHR* Electronic health record, *EPIS* Exploration, Preparation, Implementation, Sustainment, *FIT* Fecal immunochemical test, *NC* North Carolina, *PDSA* Plan-Do-Study-Act, *SCORE* Scaling Colorectal Cancer Screening through Outreach, Referral, and Engagement, *SOP* Standard operating procedures

### Preparation strategy 1: use advisory boards and workgroups

**Fig. 3:**
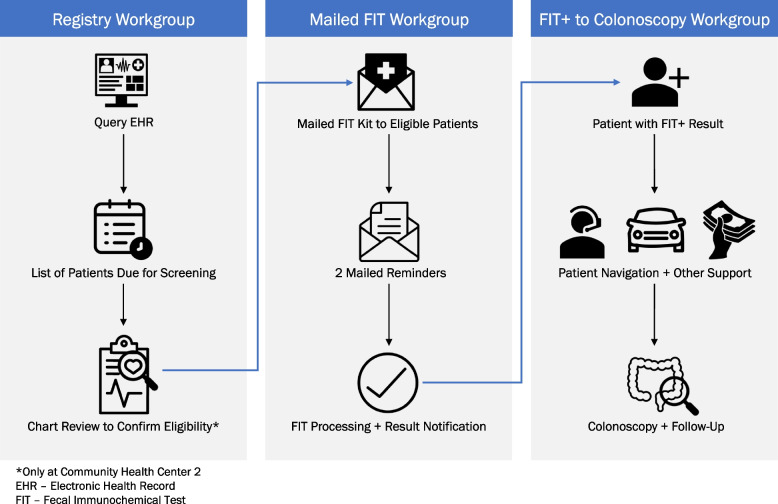
SCORE core function workgroups

Given the complexity of the planned intervention, we divided the Preparation phase work among three workgroups (Fig. 3) corresponding to the intervention’s core functions. Each workgroup served as a bridging factor, and, as such, included members of the research team and key personnel from each CHC, as well as other stakeholders (e.g., endoscopy providers). Each workgroup focused on developing one of the intervention’s core functions: (1) identifying and tracking patients due for screening who would be candidates for outreach (Registry Workgroup); (2) conducting mailed FIT outreach (Mailed FIT Workgroup); and (3) facilitating follow-up colonoscopy for FIT-positive (FIT +) patients (FIT + to Colonoscopy Workgroup). Each workgroup established a charter to aid in defining the group’s composition, objectives, and scope [28], and held regular (often weekly) working meetings that were task-oriented and focused on collaborative problem-solving.

### Preparation strategy 2: obtain formal commitments

Centralizing intervention delivery required warehousing or storing patient data in a registry outside each CHC. Regulatory requirements for data sharing and EHR access necessitated bridging factors in the form of multiple formal agreements among various entities before the intervention components could begin. For example, we obtained formal Business Associate Agreements to enable centralized mailing and processing of FITs on behalf of the CHCs and Data Use Agreements to allow the secure sharing of data. We also established formal agreements among our academic institution and the CHCs, the commercial laboratory providing and processing the FIT kits, endoscopy providers providing reduced-fee colonoscopies for the uninsured, and funding agencies.

### Preparation strategy 3: assess for readiness and identify barriers and facilitators

Workgroups were tasked with developing intervention processes, exploring inner and outer contexts of potential impact on the intervention, and identifying barriers and facilitators specific to their core function. Workgroups used quality improvement approaches to assess readiness for implementation [29]. Two specific tools included process mapping and EHR chart review. Workgroups used process mapping (also called process flow diagramming or “swimlane” diagramming) [30, 31] to clarify starting and stopping points for each workgroup’s processes as well as current processes, and to identify barriers and facilitators to executing specific process steps. To create process maps, workgroups engaged in interactive diagramming exercises, which included using sticky notes on a large wall diagram to revise steps and branching points (conditions under which process steps may differ) in screening workflows. The diagrams also helped to establish clear workflows for a population-based CRC screening outreach intervention that would complement existing visit-based screening activities and helped guide discussions about what tasks would be centralized at the outreach center or be executed at the clinical sites. Importantly, process mapping also aided in understanding how to ensure that the intervention would not impede or confuse visit-based, usual care practices. Because North Carolina CHCs operate independently, utilize different EHR systems, and vary with regard to local and regional endoscopy access, process maps also helped identify points of variation for adaptation to different contexts. Diagrams were iteratively updated during the Preparation phase, informing and informed by other implementation strategies.

Each workgroup also used patient-level EHR chart reviews to understand current processes as well as identify process variation and potential barriers to implementation. For example, the FIT + to Colonoscopy Workgroup members conducted a retrospective chart review of patients with a FIT + result in the 18 months prior to study inception to better characterize historical referral patterns and colonoscopy completion rates at each site [32]. The workgroup also used chart reviews to observe existing care patterns (without intervening) in the usual care of FIT + patients [32]. These chart reviews allowed detailed understanding and mapping of the usual care processes including documentation of abnormal screening test results, patient notification, colonoscopy referral and completion, and handling of colonoscopy results.

### Preparation strategy 4: conduct cyclical small tests of change

Cyclical small tests of change, also known as rapid Plan-Do-Study-Act (PDSA) [33] cycles, are a quality improvement strategy employing small-scale tests of change to iteratively improve care processes. In this project, work groups used PDSA cycles to refine multiple intervention processes within each core function prior to implementation. For example, the Registry Workgroup conducted a series of iterative EHR data queries for each CHC (paired with manual EHR review) to test and refine the accuracy of electronic queries that would be used to identify patients eligible to receive mailed FIT kits. Similarly, the Mailed FIT Workgroup conducted multiple “waves” of pilot FIT kit mailings (29–100 mailings per wave) to test and refine processes used to track FIT mailings, notify patients and providers of results, and refer FIT + patients for follow-up colonoscopy [34]. The FIT + to Colonoscopy Workgroup tested and refined processes for navigating patients to colonoscopy during the initial testing phase. For each PDSA cycle, the workgroup reviewed data and made iterative improvements to navigation activities, including referral processes, number of calls, content of calls, barriers addressed, and tracking of colonoscopy procedure outcomes.

### Preparation strategy 5: use data warehousing techniques

The Registry Workgroup developed a CRC screening registry that would serve as a secure, integrated database of patient-level data from different sources (CHCs, endoscopy centers, navigator calls, centralized outreach center) to facilitate efficient and accurate tracking of patients throughout the intervention. In addition to tracking patients, the registry was designed to support monitoring and reporting of effectiveness and implementation outcomes. The Registry Workgroup built the registry in REDCap, a secure, HIPPA compliant electronic data capture system hosted at our academic cancer center. Other workgroups provided input on the registry as each tested and refined their processes.

### Preparation strategy 6: develop educational materials

Two workgroups, Mailed FIT and FIT + to Colonoscopy, developed and refined patient-facing materials to promote patient understanding of CRC screening and facilitate screening. The workgroups began by creating prototype materials adapted from existing mailed FIT outreach and navigation materials [4, 19, 35–37]. Published guidance on best practices for mailed FIT outreach and navigation also informed the development of educational materials [38]. Workgroups refined materials by soliciting input from CHC clinical stakeholders at project meetings, from patients through structured interviews, and from the UNC Lineberger Community Advisory Board.

### Preparation strategy 7: fund and contract for the clinical innovation

Multiple sources funded the development and testing of the intervention, including foundation and cancer center funds and a National Cancer Institute (NCI) implementation research grant. In addition to this, Exploration phase findings suggested the need for funding to address financial barriers to colonoscopy for FIT + patients who lacked health insurance. We addressed this differently in the two CHCs based on the regional context of endoscopy service delivery. In the CHC1 region, we partnered with a large, regional gastroenterology practice that provided low-cost, fixed-fee colonoscopy to uninsured CHC patients following abnormal FIT results [39]. This partnership involved facilitating and funding an expansion of their fixed-fee program to include an additional endoscopy location adjacent to CHC1 headquarters and primary clinical site. A similar fixed-fee program was not available in the CHC2 region, so we adapted the intervention to include assisting patients with applying for financial assistance at the regional hospital where endoscopies were performed, and we developed payment systems to cover residual out-of-pocket colonoscopy-related costs. For patients with financial barriers, all costs related to the follow-up colonoscopy were covered using study funds, including colonoscopy, transportation, and other out-of-pocket costs (e.g., bowel prep supplies). We also developed processes for funding and contracting for transportation to colonoscopy for FIT + SCORE patients needing transportation assistance.

### Preparation strategy 8: develop a formal implementation blueprint

The research team integrated the Preparation phase activities and findings into a comprehensive implementation blueprint for SCORE. The blueprint included an overall project description, a manual of standard operating procedures (SOP) for the intervention with context-driven tailoring for each CHC site, patient-facing materials, timelines, and refined process maps. The implementation blueprint also included a published protocol for effectiveness evaluation [6]. SOP manuals were modeled on other published CRC screening protocols and manuals [37, 40, 41], which were revised and adapted based on findings from other Preparation phase activities. Due to the centralized nature of the SCORE intervention, the SOP was targeted to and utilized by the outreach center team performing centralized functions (rather than CHC staff) and intended as one aspect of a blueprint for intervention implementation.

## Preparation phase results

Table 3 summarizes the definitions and outcomes for each key implementation strategy used in the Preparation phase. In the text below, we highlight select findings from each of the core function workgroups. While it is beyond the scope to describe how each implementation strategy was used by each workgroup in detail, we provide examples of how workgroups used particular strategies and the associated outcomes.

### Select Registry Workgroup findings

The Registry Workgroup developed a process map detailing the steps of the EHR query process for identifying patients who are eligible and due for FIT screening. The group then conducted PDSA cycles to validate and improve the EHR data search queries, tailored to each CHC. As an example, at one CHC, an initial query yielded 135 patients (PDSA cycle 1), of whom 50% were ineligible for the intervention, with the most frequent reasons being they had already undergone a colonoscopy recently (and therefore not “due” for screening) or were no longer an active patient. We revised the query based on these results, adding or modifying parameters to better capture recent colonoscopy and active patient status. Re-running the electronic search query on the original 135 patients, and found that only 13% of identified patients were still incorrectly classified as eligible (PDSA cycle 2). This iterative process improved the identification of eligible patients. However, the misclassification rate at one of the CHC sites remained high enough that a brief manual EHR check at the individual patient level was retained for that site.

### Select Mailed FIT Workgroup findings

The Mailed FIT Workgroup developed a process map detailing the steps of the mailed FIT process, including kit assembly, mailing, and tracking returned kits (both completed and undeliverable kits). The group conducted PDSA cycles to refine outreach education materials, delivery methods, and messaging, and tested the following components of FIT outreach: (1) an initial primer letter notifying patients they are due for screening and providing the option to opt out of further contact; (2) a FIT kit mailed with a cover letter and educational materials two weeks later; (3) reminder letters sent, if needed, three and six weeks after the FIT kit was mailed; and (4) a live phone call reminder 1–2 weeks after mailing of the second reminder letter. We tested these in six small waves (cycles) in a total of 444 randomly-selected patients due for screening. Based on this testing, we adjusted the protocol (e.g., by shortening the mailing intervals for the FIT kit and reminder letters). We also decided to retain the reminder letters but discontinue the live phone reminders, which were resource intensive with minimal yield — after multiple call attempts, we reached only 46 out of 118 patients, resulting in five additional completed FIT kits.

### Select FIT + to Colonoscopy Workgroup findings

The FIT + to Colonoscopy Workgroup developed a process map detailing steps of the navigation process beginning with patient notification of abnormal results to ensuring colonoscopy results were entered into the EHR. The workgroup adapted protocols from an existing, published colonoscopy navigation program [37] to create an initial navigation protocol that included six phone calls. We tested the navigation protocol with 15 patients, refining it iteratively based on patient comments, number of call attempts and completions, and workgroup debriefings with the navigator. Ultimately, we chose to reduce the protocol from six to four standard navigation calls, due to challenges reaching patients and because we found the navigator could cover recommended navigation topics in fewer than six calls. The revised 4-call protocol included the flexibility of adapting the number of calls to the patient’s needs, recognizing that some patients are self-sufficient after one call while others require more support and more frequent contact.

Additional examples of refinements to the navigation protocols included adding more referral process details (e.g., timeframe of navigator intervening to ensure a colonoscopy referral had been initiated), procedures for monitoring completion of referrals, and developing scripts for frequently asked questions to aid the navigator in providing consistent, accurate information in response to common patient questions. We also developed protocols to ensure that patients at higher risk of complications from colonoscopy and polypectomy (e.g., those taking anticoagulant medications) received appropriate medical guidance from their primary care provider and consulting gastroenterologist prior to colonoscopy.

### Implementation blueprint

**Fig. 4:**
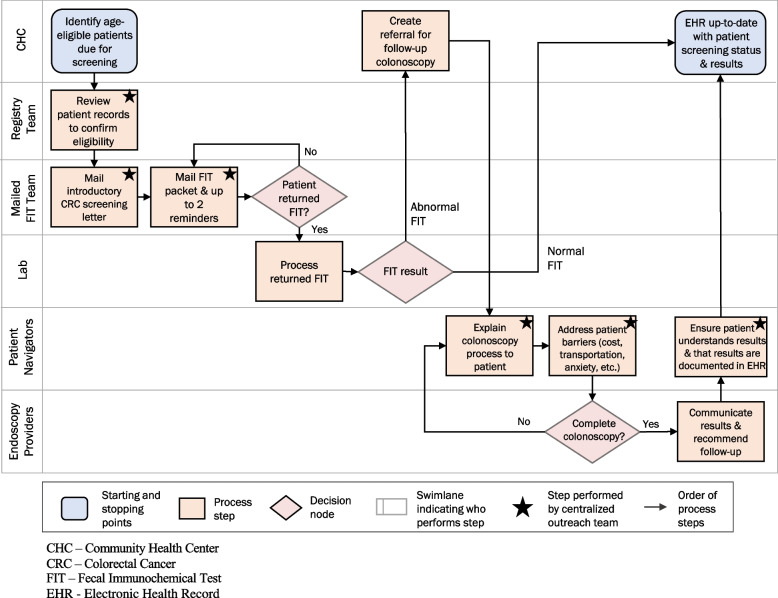
Process map providing a high-level overview of the SCORE intervention

**Fig. 5:**
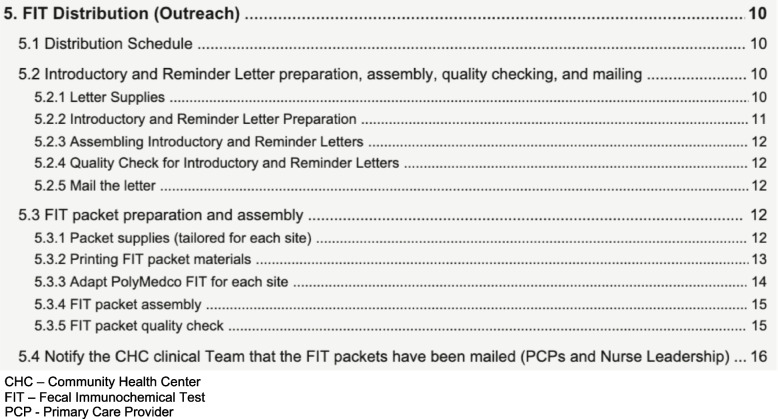
SCORE intervention standard operating procedures (SOP) table of contents excerpt: FIT Distribution

Preparation phase workgroup activities culminated in a comprehensive implementation blueprint to guide intervention implementation. This blueprint included detailed process maps from each workgroup, an overview process map integrating the individual workgroup process maps (Fig. 4), as well as an SOP manual (see Fig. 5 for the SOP excerpt). As shown in Fig. 4, each row or “swimlane” corresponds to a different entity engaged in the process, with separate swimlanes for the activities performed by each of the following SCORE stakeholders: CHC staff and providers, centralized outreach center (registry team, mailed FIT team, and navigators), FIT processing laboratory, and endoscopy providers. The SOP manual (Fig. 5) includes detailed protocols for how to maintain a registry of patients due for screening, mail patient-facing materials and FIT kits, track FIT returns, enter results into the EHR, notify providers and patients of FIT results, and provide navigation services to FIT + patients.

## Discussion

We have described how multiple implementation strategies were used during the Exploration and Preparation phases of SCORE, a CRC screening implementation project. The strategies used during Exploration led to consensus on selection of the EBPs needed to fill a screening gap while strategies used in the Preparation phase culminated in a comprehensive implementation blueprint. In a recent systematic review of EPIS, Moullin and colleagues found that most published implementation studies have focused on the Implementation phase of EPIS and that there is a paucity of empiric research addressing the Exploration and/or Preparation phases [11]. The authors noted other literature suggesting that in-depth planning during the Exploration and Preparation phases was “infrequent although critical” [11]. Thus, our study adds to a limited body of in-depth empiric description of an approach to these early phases of implementing a multilevel outreach intervention in health care.

Our study also addresses bridging factors, a relatively new and understudied component of the EPIS framework [9]. We developed bridging factors (i.e., relational ties and formal arrangements) that facilitated implementation by connecting the stakeholders and organizations comprising the inner and outer contexts of the intervention components. Lengnick-Hall and colleagues argue that identifying bridging factors is particularly important for implementation initiatives, such as ours, that are multilevel and require coordination across stakeholders at the level of the inner and outer contexts [9]. Consistent with our project’s complexity, several of our implementation strategies yielded bridging factors (see Fig. 2), including building a coalition, using advisory boards or workgroups, and obtaining formal commitments. Each of these strategies promoted and supported communication, interaction, and the exchange of resources among diverse organizations and stakeholders [9, 42].

Our study was guided by the Expert Recommendations for Implementing Change (ERIC) compilation, in which Powell and colleagues aimed to generate a comprehensive list of implementation strategies for research and practice [10]. In SCORE, we applied the ERIC compilation to the Exploration and Preparation phases of a large implementation effort, describing (a) how each strategy was operationalized in this implementation context, and (b) the proximate results or outcomes.

We found centralization to be critical to developing the SCORE intervention in this context. This finding is consistent with arguments put forth by Birken, Leeman, and others that organizational theories, which explain interactions between organizations and their external context, can be used in planning for implementation [43, 44]. Centralization may be particularly appropriate in contexts such as these rural CHCs, which have limited staff resources with which to deliver interventions with fidelity, limited control over external organizations, and low patient volume, resulting in high transaction costs and motivating the outsourcing of implementation activities to a centralized entity with dedicated and specialized resources, per transaction cost economics theory [45]. Although resource dependency theory suggests that CHCs may become dependent on the centralized entity and potentially unable to sustain EBI implementation in the absence of this relationship [45], dependency was counteracted through integration into the implementation team.

Support for centralization also comes from experience with and publications of existing organized mailed FIT programs, which have been implemented mainly in very large, integrated health networks or in national programs [46–49]. Our implementation context contrasts sharply with these settings in consisting of small, independent CHCs with limited capacity to develop, test, refine, and deliver the components of a multilevel screening outreach intervention. Furthermore, unlike large integrated systems, our outer context was also characterized by variation in commercial laboratories, EHR systems, FIT kit brands, and payer policies, as well as in endoscopy providers and facility types available for follow-up of abnormal FITs. To address this organizational fragmentation, we developed a cancer center-based intervention hub that could functionally emulate some of the characteristics of large health systems by standardizing outreach processes and FIT materials as well as navigator protocols.

Similar to Cook and colleagues, we identified strategies that might be considered candidates for future inclusion in the ERIC compilation [50]. The first such strategy is “review and appraise current evidence regarding effective interventions.” Considering this as an explicit strategy is supported by research showing that systematic reviews and associated guidelines have relatively short half-lives, often needing to be updated within a few years of publication [51]. In our case, during Exploration, we became aware that the widely-cited Centers for Disease Control and Preventions’ Community Preventive Services Task Force (CPSTF) recommendations for interventions to increase CRC screening did not reflect several more recently published intervention trials, particularly around mailed stool test outreach and patient navigation [52]. We therefore chose to conduct our own synthesis of high-quality randomized trials of interventions to increase CRC screening and found robust evidence that mailed stool test outreach was consistently and substantially effective [13]. This review helped guide our choice of selecting approaches to the problem of CRC screening underuse.

In this paper, we included our evidence review and appraisal activity as a modification of the “conduct local needs assessment” strategy. However, understanding the depth, breadth, and nuances of intervention literature is critical in selecting interventions, and also, we believe, qualitatively distinct. While formal systematic reviews are time-consuming and not feasible in most implementation contexts, we suggest implementers seek current evidence about relevant EBPs during intervention planning and development.

A second strategy we used that could be considered a distinct implementation strategy is “use quality improvement and systems science tools to map and model complex processes.” Quality improvement practices aim to systematically identify and address gaps in practices and outcomes [53], typically using tools such as process mapping and Plan-Do-Study-Act (PDSA) cycles. Systems science examines complex systems in any scientific field that are characterized by dynamic and interactive behavior [24, 54, 55]. Systems science has developed approaches and techniques that can both help decision makers understand complex systems and guide implementation within a complex and dynamic context. Wheeler and colleagues have described how systems science approaches can be used to guide the selection and implementation of multilevel CRC screening interventions [56].

In this paper, we describe the use of one systems science tool, process mapping, to facilitate communication among staff, develop a shared understanding of implementer roles and responsibilities, and identify opportunities to standardize workflows. This allowed us to tailor protocols to the varying CHC context as needed and led to improved efficiency by uncovering unnecessary complexity, redundancy, gaps, and bottlenecks. We also used the process maps in planning our economic evaluation, to identify resources (costs) associated with implementing the intervention moving forward [57].

## Conclusions

We describe strategies employed during the Exploration and Preparation phases of our multilevel intervention to enhance colorectal cancer screening in community health centers. The implementation strategies used during these first two phases yielded a comprehensive blueprint for the third phase, Implementation. Through coalition building and the use of workgroups, stakeholder partners developed consensus about and ownership of the intervention and its implementation. We identified two implementation strategies that could be considered candidates for addition to the ERIC compilation: review and appraise current evidence regarding effective interventions and use systems science tools to map and model complex processes. Our study may be useful both to implementation practitioners seeking exemplars for operationalizing strategies in early phases of implementation in healthcare and to implementation scientists, who can draw lessons from this type of applied research to refine their implementation strategy typologies and frameworks. The Implementation phase of our study will test our conclusions and inform sustainability efforts, as described in our protocol paper [6]. In the fourth phase, Sustainment, we anticipate revisiting strategies as we engage stakeholders in scale-up and sustainment planning. Sustainment will likely require additional support for CHCs, for example, Health Resources and Services Administration (HRSA) funding [58] for mailed FIT and navigation activities currently supported by study funding but not yet reimbursed in current payment models. Future publications will describe the Implementation and Sustainment phases of this project. 

### Supplementary Information

**Additional file 1.** Adherence to reporting guidelines. Description of project adherence to reporting guidelines as outlined in Proctor, E.K., Powell, B.J. & McMillen, J.C. Implementation strategies: recommendations for specifying and reporting. Implementation Sci 8, 139 (2013).

## Data Availability

The datasets used and/or analyzed during the current study are available from the corresponding author on reasonable request.

## Abbreviations

ACCSIS: Accelerating Colorectal Cancer Screening and Follow-up through Implementation Science
BAA: Business Associate Agreement
CHC: Community health center
CHC1: Community health center 1
CHC2: Community health center 2
CRC: Colorectal cancer
DUA: Data Use Agreement
EBP: Evidence-based practice
EHR: Electronic health record
EPIS: Exploration, Preparation, Implementation, Sustainment
ERIC: Expert Recommendations for Implementing Change
FIT: Fecal immunochemical test
FIT +: FIT positive
FQHC: Federally Qualified Health Centers
NC: North Carolina
NCI: National Cancer Institute
PDSA: Plan-Do-Study-Act
SCORE: Scaling Colorectal Cancer Screening through Outreach, Referral, and Engagement
SOP: Standard operating procedures
UNC: University of North Carolina at Chapel Hill
