# Supplementary material for: Implementation strategies in the Exploration and Preparation phases of a colorectal cancer screening intervention in community health centers
Source: Implement Sci Commun. 2023 Sep 20;4:118. doi: 10.1186/s43058-023-00485-5 (PMC10512568; doi:10.1186/s43058-023-00485-5)
Supplement: Supplementary file 1 — Additional file 1. Adherence to reporting guidelines. Description of project adherence to reporting guidelines as outlined in Proctor, E.K., Powell, B.J. & McMillen, J.C. Implementation strategies: recommendations for specifying and reporting. Implementation Sci 8, 139 (2013). [file 43058_2023_485_MOESM1_ESM.docx]

Manuscript: Implementation Strategies in the Exploration and Preparation Phases of a Colorectal Cancer Screening Intervention in Community Health Centers, by Ferrari et al.

Reporting Guideline: Proctor, E.K., Powell, B.J. & McMillen, J.C. Implementation strategies: recommendations for specifying and reporting. Implementation Sci 8, 139 (2013). https://doi.org/10.1186/1748-5908-8-139

Additional File. Description of project adherence to reporting guidelines

| Prerequisite | Requirements |
| --- | --- |
| 1) Name it | We named a group of strategies drawn/adapted from the Expert Recommendations for Implementing Change (ERIC) compilation.^1^ |
| 2) Define it | We define and operationalize each strategy in Tables 1 and 3. |
| 3) Specify it |  |
| a) The actor | We identify the actor for each strategy throughout the narrative section, and particularly in the core function workgroup activity descriptions. |
| b) The action | We describe the specific actions taken to implement the strategy throughout the text, and particularly in the core function workgroup activity descriptions.. |
| c) Action target | We specify the action targets for each strategy throughout the text, and particularly in the core function workgroup descriptions. |
|  | Identify unit of analysis for measuring implementation outcomes – not applicable |
| d) Temporality | We describe the strategies used during the Exploration and Preparation phases of the Exploration, Preparation, Implementation, Sustainment (EPIS) framework (cite). |
| e) Dose | We describe the dose for applicable strategies. For example, for the strategy of ‘use advisory board and workgroups’, we describe how often groups met to work on the intervention implementation. |
| f) Implementation outcome affected | Because this is early phase work, we identify and describe how strategies were used to improve contextual fit of the core functions of the intervention. Implementation outcome measurement is not within the scope of this paper and will be reported in future publications. |
| g) Justification | We selected strategies from the ERIC compilation, commonly used in implementation science as it’s an expert-devised compilation developed using a modified Delphi method for consensus.^1^ We also chose strategies together with our partners based on practical application in their context. |

^1^Powell BJ, Waltz TJ, Chinman MJ, Damschroder LJ, Smith JL, Matthieu MM, et al. A refined compilation of implementation strategies: results from the Expert Recommendations for Implementing Change (ERIC) project. Implement Sci. 2015;10:21.

^2^Aarons, G.A., Hurlburt, M. & Horwitz, S.M. Advancing a Conceptual Model of Evidence-Based Practice Implementation in Public Service Sectors. *Adm Policy Ment Health* **38,**4–23 (2011). https://doi.org/10.1007/s10488-010-0327-7
